# Salvianolic Acid A Inhibits PDGF-BB Induced Vascular Smooth Muscle Cell Migration and Proliferation While Does Not Constrain Endothelial Cell Proliferation and Nitric Oxide Biosynthesis

**DOI:** 10.3390/molecules17033333

**Published:** 2012-03-14

**Authors:** Lan Sun, Rui Zhao, Li Zhang, Tiantai Zhang, Wenyu Xin, Xi Lan, Chao Huang, Guanhua Du

**Affiliations:** Beijing Key Laboratory of Drug Target and Screening Research, Institute of Materia Medica, Chinese Academy of Medical Science and Peking Union Medical College, 1 Xian Nong Tan Street, Beijing 100050, China

**Keywords:** salvianolic A, vascular smooth muscle cell, proliferation, migration, re-endothelialization

## Abstract

Proliferation and migration of vascular smooth muscle cells (VSMCs) are critical events in the initiation and development of restenosis upon percutaneous transluminal coronary angioplasty (PTCA). Polyphenols have been suggested to ameliorate post-angioplasty restenosis. Salvianolic A (SalA) is one of the most abundant polyphenols extracted from salvia. In this study, we investigated the effect of salvianolic A (SalA) on the migration and proliferation of VSMCs. We found a preferential interaction of SalA with cellular systems that rely on the PDGF signal, but not on the EGF and bFGF signal. SalA inhibits PDGF-BB induced VSMC proliferation and migration in the concentration range from 0.01 to 0.1 μM. The inhibition of SalA on VSMC proliferation is associated with cell cycle arrest. We also found that SalA inhibits the PDGFRβ-ERK1/2 signaling cascade activated by PDGF-BB in VSMCs. In addition, SalA does not influence the proliferation of endothelial cells, the synthesis of NO and eNOS protein expression. Our results suggest that SalA inhibits migration and proliferation of VSMCs induced by PDGF-BB via the inhibition of the PDGFRβ-ERK1/2 cascade, but that it does not constrain endothelial cell proliferation and nitric oxide biosynthesis. Thus, the present study suggests a novel adjunct pharmacological strategy to prevent angioplasty-related restenosis.

## 1. Introduction

The increased proliferation and migration of vascular smooth muscle cells (VSMCs) are critical events in the initiation and development of restenosis upon percutaneous transluminal coronary angioplasty (PTCA) [1]. Both of these events can be induced by cytokines and growth factors, such as platelet-derived growth factor (PDGF), fibroblast growth factor (FGF), and epidermal growth factor (EGF) [2]. Platelet-derived growth factor-BB (PDGF-BB), which activates the PDGF receptor (PDGFR)-β on VSMCs in the media, is one of the most potent mitogens and chemoattractants for vascular smooth muscle cells (VSMCs) and plays the central role in provoking restenosis [3]. Binding of PDGF-BB to the PDGF-Rβ, activates PDGF-Rβ and then initiates a multitude of biological effects through the activation of mitogen-activated protein kinases(MAPK), including extracellular signal-regulated kinase (ERK), c-Jun NH2-terminal kinase (JNK), Akt and p38 MAPK (p38) that contribute to VSMC proliferation, migration, and collagen synthesis [4,5]. Activation of the MAPK is required for mitogenic signaling through a number of tyrosine kinase growth factor receptors.

**Figure 1:**
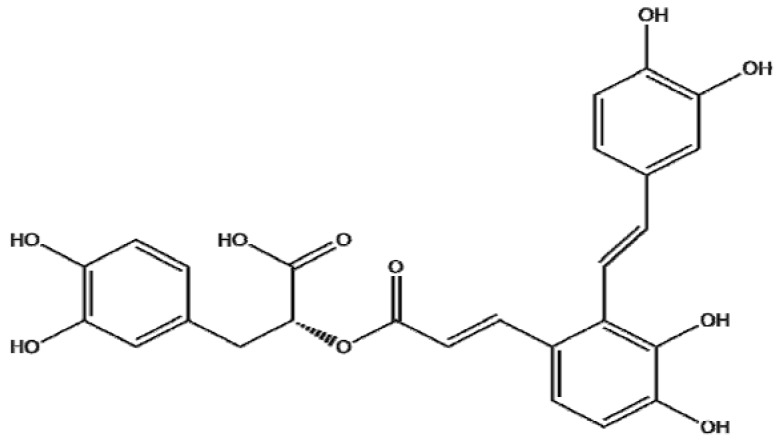
The chemical structure of salvianolic acid A (SalA). Molecular formula: C_26_H_22_O_10_, Molecular weight: 494.45.

Salvianolic extracts (commonly named “Danshen” in China) are among the most frequently used herbal medicinal products in China. According to Traditional Chinese Medicine, Danshen can be used to promote blood flow and to resolve blood stasis. Therefore, it is widely prescribed to patients with angina pectoris, hyperlipidemia, and acute ischemic stroke [6]. Salvianolic acids are the most abundant water-soluble compound extracted from salvia. Among salvianolic acids, salvianolic A (SalA) is one of the most abundant polyphenols. The structure of SalA is shown in Figure 1.

Polyphenols have been suggested to prevent post-angioplasty restenosis via the inhibition of VSMC migration and proliferation, but not constraining re-endothelialization. Recently, it has been found that salvianolic B inhibits VSMC proliferation and migration by suppressing the expression level of CXCR4 receptor [7]. Therefore, we investigated the effects of SalA on the migration and proliferation of VSMCs *in vitro*. We analyzed its impact on cell proliferation induced by different growth factors as well as on growth factor receptor activity and explored the underlying mechanisms. In addition, the influence of SalA on re-endothelialization was also examined.

## 2. Results and Discussion

Danshen extracts, such as salvianolic A belongs to the most popular and widely used herbal remedies in China. The efficacy of SalA for the adjunctive treatment of microcirculation protection [8], cerebral protection [9], and myocardial protection [10] has been well documented. Our study indicates that SalA inhibits VSMC proliferation and migration, but does not constrain endothelial cell proliferation and the biosynthesis of nitric oxide (NO), major protective and therapeutically valuable processes upon vessel injury. In addition, our study suggests that the anti-proliferative and anti-migratory action of SalA depends on the cell type and is not a general cell division-inhibiting or toxic effect. And at the same time, Sal A demonstrates an inhibitory effect on the activation of PDGFR/ERK cell signaling pathway. To our knowledge, this study is the first one to investigate the potential role of SalA in VSMC proliferation and migration induced by PDGF-BB, which may provide novel insights into its contribution to the therapy and prevention of stent restenosis.

### 2.1. Sal A Inhibits PDGF-BB Induced VSMC Proliferation and Migration

**Figure 2:**
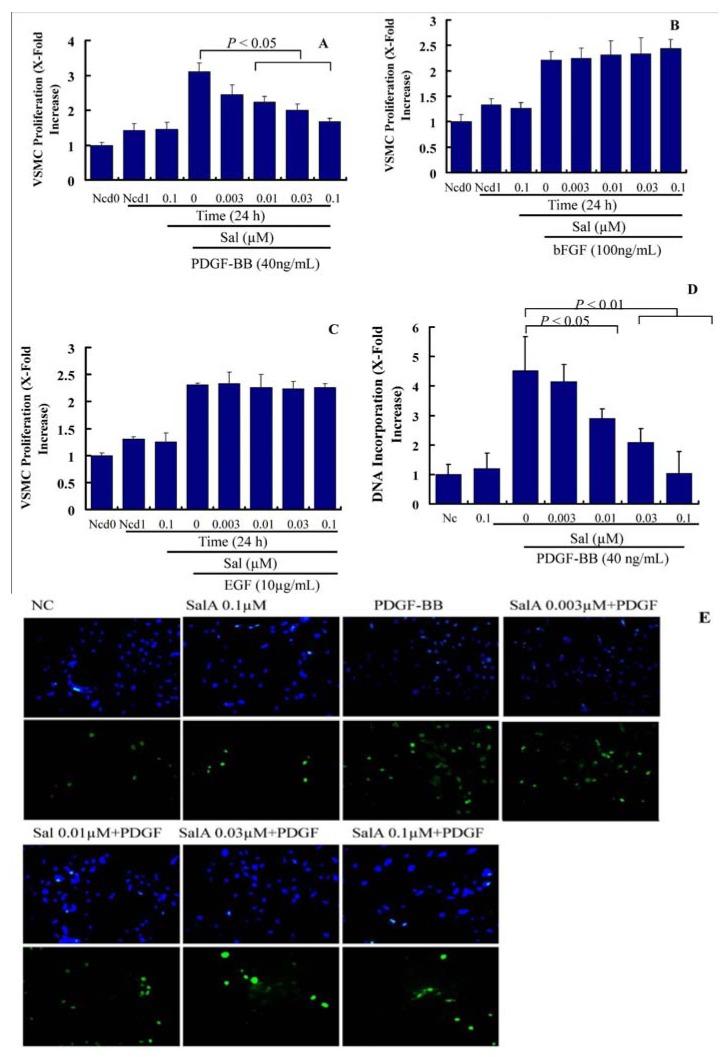
SalA inhibibits PDGF-BB-induced VSMC proliferation. Confluent VSMCs (starved for 24 h in FCS-free DMEM) were treated with SalA at different concentrations (0–0.1 μM) for 2 h and were then incubated in the presence of PDGF-BB (40 ng/mL), EGF (10 μg/mL), or bFGF (100 ng/mL) for another 24 h. (**A**–**C**) Crystal violet staining was used to detect VSMC proliferation. Ncd0: Initial cell number. Ncd1: Cell number after treatment with PDGF-BB (**A**), bFGF (**B**), or EGF (**C**) for 24 h. Proliferation is expressed as x-fold increase compared to Ncd0; (**D**) BrdU (10 μM) was added for the last 5 h of treatment. Incorporation of BrdU into newly synthesized DNA was determined; (**E**) After incubation with mouse anti-BrdU antibody and isotype-matched fluorescein isothyiocyanate (FITC)-conjugated anti-rat IgG1 secondary antibody (bottom), DAPI was employed to detect nuclei (upper).Data are expressed as mean ± SEM and are representative of three independent experiments.

Migration and proliferation of VSMCs, leading to intimal thickening, cause the pathogenesis of stent restenosis. Both of these events can be induced by cytokines and growth factors. In the present study, we measured the effects of SalA on PDGF-, EGF-, and bFGF-induced VSMC proliferation. As shown in Figure 2A, treatment with PDGF-BB for 24 h led to a ~2.2-fold increase in VSMC proliferation. Compared with PDGF-BB alone, pretreatment with SalA for 2 h suppressed PDGF-induced VSMC proliferation in a concentration-dependent manner in the concentration range from 0.01 to 0.1 μM, but has little effect on VSMC proliferation induced by EGF and bFGF. This suggests the specific inhibition of SalA on PDGF-induced but not on EGF-, and bFGF-induced VSMC proliferation, thus indicating that SalA selectively acts on the PDGF-induced signaling system.

As a next step, we tested the influence of Sal A on the PDGF-BB-induced cellular DNA synthesis by using BrdU incorporation assay in VSMCs. As shown in Figure 2B,C, treatment with PDGF-BB for 24 h led to a ~4.5-fold increase in the DNA incorporation rate in VSMCs. Compared with PDGF-BB alone, pretreatment with SalA for two hours concentration-dependently blunted PDGF-induced DNA incorporation by 45.9% (*p* < 0.05), 69.2% (*p* < 0.01) and 99.13% (*p* < 0.01), respectively. These results confirm that SalA acts on the PDGF-induced VSMC proliferation.

The effect of SalA on VSMC migration, which represents a crucial process in the pathogenesis of neointima formation, was also investigated. In the wound closure/scratch assay, which determines the non-directional migratory activity due to the loss of neighboring cells, we observed a VSMC migration-inhibiting activity of SalA at a concentration range between 0.003 and 0.1 μM.

**Figure 3:**
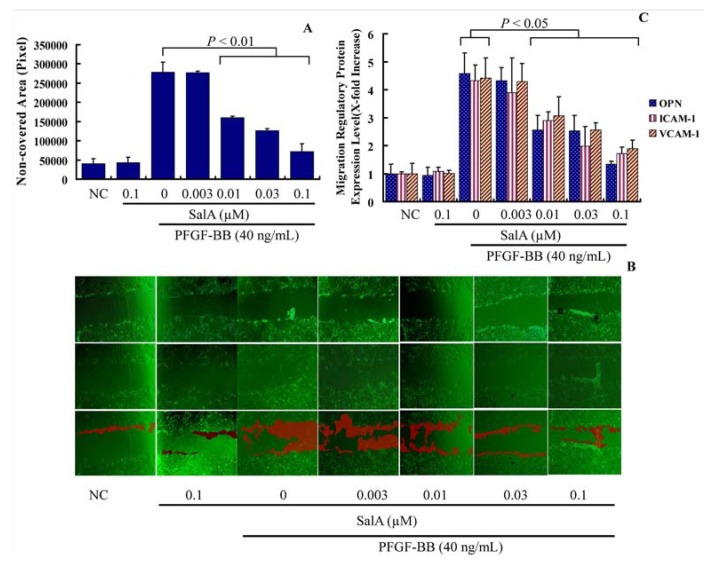
SalA inhibits adhesion molecule expression and VSMC migration induced by PDGF-BB. Confluent VSMCs (starved for 48 h in FCS-free DMEM) were treated with SalA at different concentrations (0.003–0.1 μM) for 2 h and then were incubated with PDGF-BB for another 12 h. The migration/growth medium was DMEM without L-arginine containing 5 mM hydroxyurea to prevent VSMC proliferation. Four different fields of migration were photographed with a video camera system using Image Pro Plus 5.1 software (Media Cybernetics, Silver Spring, MD, USA) at the intersection of the previously marked line and wound edge before and after treatment with PDGF-BB. The migration activity was expressed as the change in covered area. Samples were run in triplicate in three independent experiments. (**A**) Change in covered area; (**B**) The upper, middle, and bottom images represent the beginning point, end point, and merged images, respectively. The red shadow represents the change in covered area; (**C**) Levels of OPN, VCAM-1, and ICAM-1, as determined by ELISA. The relative level of these proteins is expressed as x-fold increase compared to the normal control group for three independent experiments.

As is shown in Figure 3A, t reatment with PDGF-BB for 12 h led to a ~6.9-fold increase in the basal migration of VSMCs. Compared with PDGF-BB alone, pretreatment with SalA at 0.03–0.1 μM for two hours caused a significantly reduction of cell migration (Figure 3A). Then, the expression levels of several migration regulatory proteins, including osteopontin (OPN), intercellular adhesion molecule 1 (ICAM-1) and vascular cell adhesion molecule 1 (VCAM-1) were also detected. As is shown in Figure 3B, PDGF-BB significantly induced the expression level of ICAM-1, VCAM-1, and OPN. Pretreatment with SalA reduced the expression level of OPN, VCAM-1 and ICAM-1 nearly to a normal level (*p* < 0.05), thus suggesting that SalA inhibits the migration of VSMCs induced by PDGF-BB via suppressing the expression of migration-related proteins in these cells.

Upon arterial injury or in atherosclerotic lesions, VSMCs are exposed to PDGF [11,12]. The increase of PDGF after arterial injury positively correlated to neointimal and cellular proliferation [13,14,15,16], demonstrating a closely association between proliferation and migration of VSMCs and PDGF. Our results indicate the preferential interaction of SalA with cellular systems that relies on the PDGF signal, which is demonstrated by the specific inhibiting effects on PDGF-BB induced VSMC proliferation but not on EGF- and bFGF-induced VSMC proliferation. This is in accordance with the result that SalA inhibits PDGF-BB-induced cellular DNA synthesis. Furthermore, SalA shows the concentration-dependently inhibiting effects on PDGF-BB induced VSMC migration. Therefore, SalA is a potentially important therapeutic strategy for the treatment of stent restenosis and other cardiovascular diseases.

### 2.2. The Inhibition of on VSMC Proliferation by SalA is Associated with Cell Cycle Arrest

**Figure 4:**
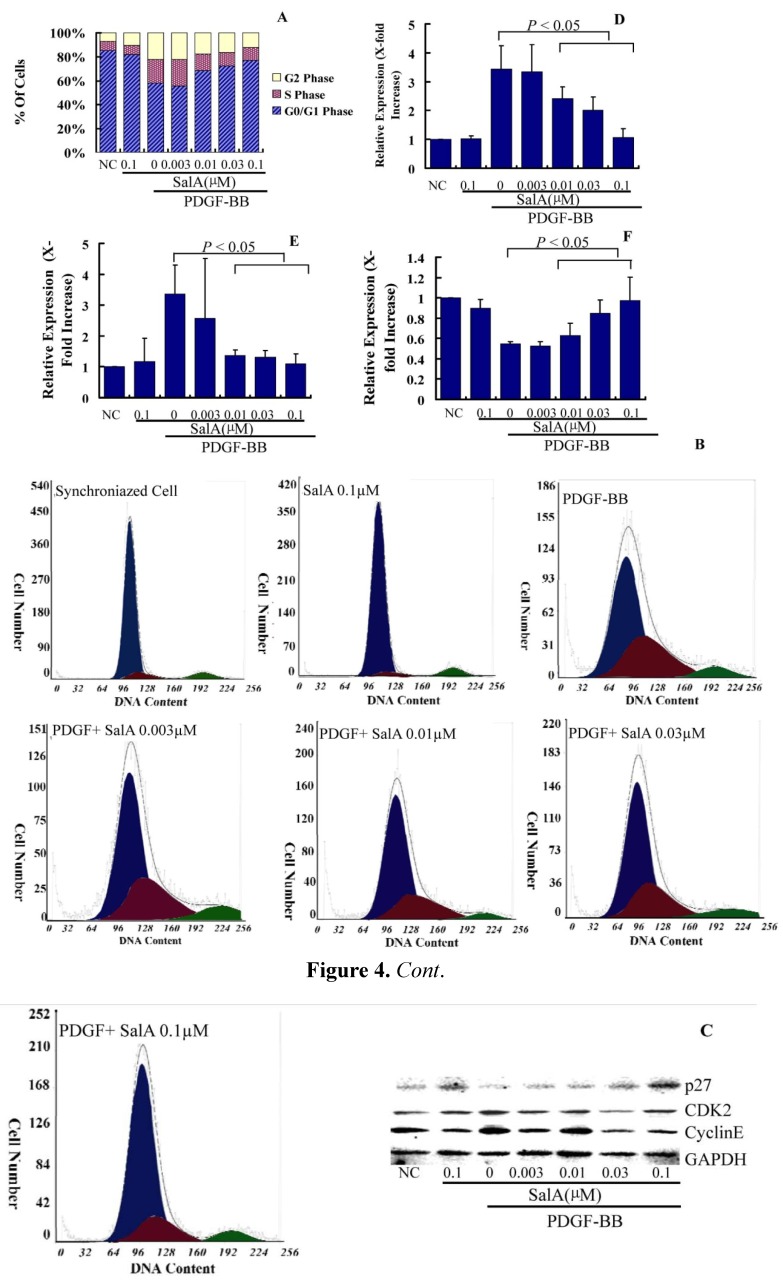
SalA induced G0/G1 phase arrest in cell cycle progression. VSMCs were pre-incubated in the presence or absence of SalA in serum-depleted medium for 24 h. The cells were then stimulated with 40 ng/mL of PDGF-BB for 24 h. (**A**,**B**) Individual nuclear DNA content was determined by measuring fluorescence intensity of incorporated propidium iodide. Each value is derived from a representative experiment where data from at least 10,000 events were obtained. The data are expressed as mean values from 3 independent experiments. The blue, red and green shadows represent the G0/G1 phase, S phase and G2 phase, respectively; (**C**–**E**) Western blot analysis was performed with antibodies specific for cyclin D1 (**D**), CDK2 (**E**) and p27 (**F**). GAPDH was used for normalization. The relative level of these proteins is expressed as x-fold increase compared to that of the normal control group.

In addition, a concentration-dependent increase in the cell population in the G0/G1 phase of the cell cycle and a concomitant decrease in the cell population in the S phase of the cell cycle were observed when VSMCs were pretreated with SalA (0.003–0.1 μM) to PDGF-BB-stimulated VSMCs. As is shown in Figure 4, the serum-deprivation of VSMCs for 24 h resulted in an approximately 7.62% synchronization of the cell cycle in the S phase. PDGF-BB treatment of the VSMCs for 24 h increased the S phase population to 19.58%. Pretreatment with SalA at the concentration of 0.01, 0.03 and 0.1 μM significantly reduced the cell population in the S phase to about 13.93 (*p* = 0.09), 11.36 (*p* < 0.01), and 10.9% (*p* < 0.01) respectively, in PDGF-BB-stimulated cells (Figure 4A,B). However, PDGF-BB treatment of the VSMCs for 24 h reduced the G0/G1 phase population to 58.28%. Pretreatment with SalA at the concentration of 0.01, 0.03 and 0.1 μM increased the cell population in the G0/G1 phase to about 68.63% (*p* < 0.01), 72.73% (*p* < 0.01) and 77.14% (*p* < 0.01). This indicates that the inhibition of SalA on VSMC proliferation was associated with cell cycle arrest in the G0/G1 phase. In addition, the highest concentration of SalA alone did not markedly alter the ratio of cells in G0/G1, S or G2 phase, which indicates that SalA alone does not affect the cell cycle of quiescent VSMCs and that pretreatment of SalA likely acts by impairing the response capacity of PDGFRβ to PDGF-BB stimulation.

Cell cycle progression is strictly controlled by the positive and negative regulators that act at checkpoints throughout the cell cycle. p27kip1 (p27) is an important negative regulator of the protein kinase CDK2/cyclin E, and can block the cell cycle at G0/G1 phase [17]. The levels of p27 are high in the G0/G1 phases of cell cycle. p27 is rapidly degraded when VSMC is stimulated by PDGF-BB, and thus CDK2/cyclin E promote cell to overcome the restriction point of G1 and cause the cell cycle progression.

Because SalA induced cell cycle arrest in G0/G1 phase, we then detected the expression level of G0/G1 phase regulatory proteins. As is shown in Figure 4C, treatment with PDGF-BB in VSMCs for 24 h led to a 3.43- and 3.99-fold increase in the relative expression level of CDK2 and Cyclin E, respectively, while the expression level of p27 was reduced about 50% in VSMCs. Pretreatment with SalA at the concentration of 0.01, 0.03 and 0.1 μM significantly reduced the expression level of CDK2 (60%, 62%, 71%) and cyclin E [40%, 50% and 72% (*p* < 0.05)], while it increased the expression level of p27 by 15.12%, 54.29% and 77.38% by comparing with PDGF-BB stimulated VSMCs (*p* < 0.05). Therefore, SalA may regulate the expression of G0/G1-checkpoint proteins, which was in accordance with the results of cell cycle analysis. Thus, the inhibition of VSMC proliferation by SalA is associated with cell cycle arrest.

### 2.3. SalA Inhibits the PDGFRβ-ERK1/2 Signaling Cascade Activated by PDGF-BB in VSMCs

During endothelial injury after angioplasty or on the early stage of atherosclerosis, the PDGF-R/MAPK signaling pathway is upregulated. To further delineate the cellular and molecular mechanisms underlying SalA-induced growth inhibitionof VSMCs, we evaluated the effect of SalA on PEGFR and MAPK signaling cascades. The binding of PDGF-BB to the PDGF receptor (PDGF-R) leads to PDGF-R phosphorylation on multiple tyrosine residues at the receptor, which in turn potently activates MAPK by triggering RAS-RAF activation in VSMCs, resulting in cell cycle progression [18]. Therefore, the inhibition of VSMC proliferation and migration by regulation of the PDGF signaling pathway could be a key pharmacological strategy for the prevention of stent restenosis and atherosclerosis.

**Figure 5:**
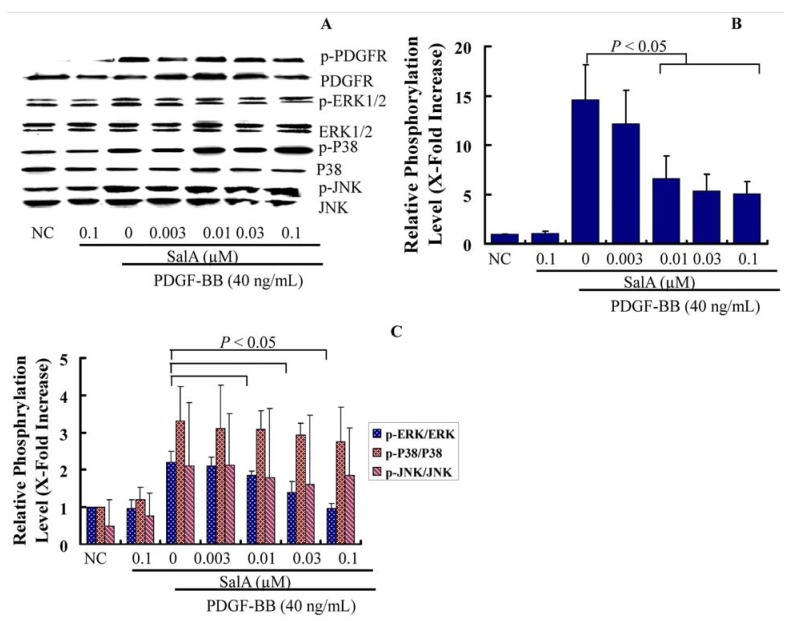
SalA inhibits the PDGFRβ-ERK1/2 signaling cascade activated by PDGF-BB in VSMCs. Confluent VSMCs (starved for 48 h in FCS-free DMEM) were treated with SalA at different concentrations (0.003–0.1 μM) for 2 h and then were incubated in the presence of PDGF-BB for another 30 min. The cells were then lysed, and proteins were analyzed by using 12% SDS-PAGE. Western blot analysis was performed to detect the phosphorylation of PDGFR-β and ERK1/2. (**A**) Representative data from 3 different experiments are presented; (**B**,**C**) The phosphorylation level of these proteins is expressed as x-fold increase compared to that of normal control group. Data shown are the mean ± SEM from three independent experiments.

In the present study, after pretreatment with SalA for two hours, VSMCs were stimulated with PDGF-BB for 30 min, and the phosphorylation status of PDGFR-β, ERK, c-Jun NH2-terminal kinase (JNK), and p38 MAPK was measured by Western blotting using antibodies that identify the active (phosphorylated) forms of these kinases. The results showed that SalA markedly inhibited MEK-ERK1/2 activation by PDGF-BB in a concentration-dependent manner in VSMCs, which was associated with a reduction of PDGFRβ phosphorylation, while there were no changes in the phosphorylated forms of JNK, p38 (Figure 5), thus indicating that the protein of EKR1/2 may be related to the antiproliferative activity of SalA. This is in accordance with another study in which salvianolic acids were found to inhibit the proliferation of rat aortic smooth muscle A10 cells stimulated by homocysteine through the blockage of the PKC/p44/42 MAPK dependent pathway [19]. ERK1/2 was induced after arterial injury in rats [20], and either an ERK1/2 inhibitor [21] or the gene transfer of an ERK1/2 dominant-negative mutant [22] can suppress VSMC proliferation and block neointimal formation in balloon-injured arteries. Hence, our study suggests that SalA can inhibit VSMC proliferation, not only by the effects on PDGFR, but also on ERK1/2.

### 2.4. Sal A Does Not Constrain Endothelial Cell Proliferation and NO Biosynthesis

**Figure 6:**
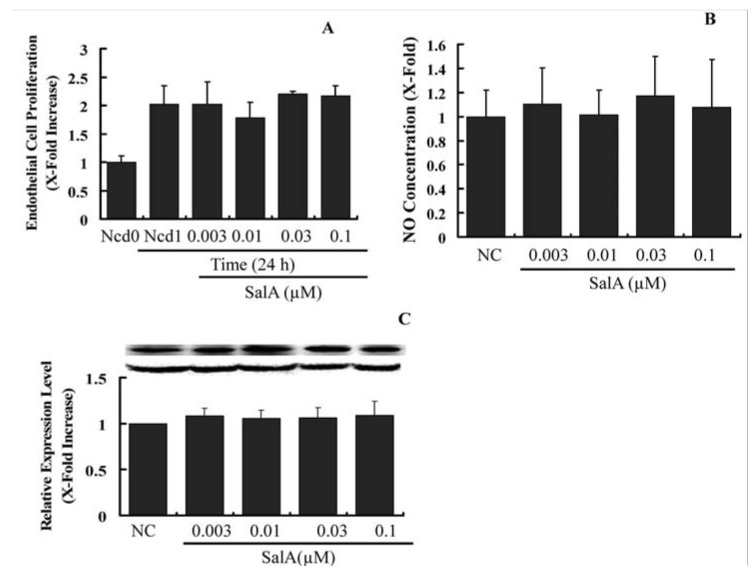
Sal A does not constrain endothelial cell proliferation and does not influence eNOS expression. Confluent HUVECs (starved for 24 h in FCS-free DMEM) were treated with SalA at different concentrations (0.003–0.1 μM) for 24 h. Crystal violet staining was used to detect cell proliferating activity (**A**); Supernatants were then collected and NO levels were measured by using the Griess reagent. Concentration was adjusted according to cell number (**B**); Western blot analysis was performed to detect the relative expression level of eNOS in HUVECs. GAPDH was used for normalization (**C**). The relative level of the cell number and NO concentration is expressed as x-fold increase compared to that of the normal control group. Data shown are the mean ± SEM from three independent experiments.

Upon arterial injury, endothelial cell growth and the expression level of important vasodilator, endothelial nitric oxide (NO) synthase (eNOS) is reduced [23]. This hampers the process of re-endothelialization, which is a major protective and thus a therapeutically valuable process upon vessel injury [24]. Hence, we were interested in to find out whether SalA influence the endothelial cell proliferation, the expression of eNOS and the cellular level of NO. As shown in Figure 6, Sal A neither altered endothelial cell proliferation nor reduced the level of eNOS (Figure 6B) and NO (Figure 6C), suggesting that the proliferation of endothelial cell and the crucial endothelial regulators of vasodilatation are not affected by the SalA. However, whether SalA may hamper the re-endothelialization upon vessel injury or nor need to be further verified *in vivo*. Our results indicate that the anti-proliferative and anti-migratory action of SalA depends on the cell type and is not a general cell division-inhibiting or toxic effect.

## 3. Experimental

### 3.1. Cell Culture

The experimental protocol conformed to the Guide for the Care and Use of Laboratory Animals published by the US National Institutes of Health (NIH Publication No. 85–23, revised 1996) [25] and was approved by the Animal Studies Committee of Peking Union Medical College. Human umbilical vein endothelial cells (HUVECs, cat# CRL-1730, ATCC) were cultured as previously described [26]. VSMCs derived from the carotid arteries of male Sprague-Dawley rats were cultured as previously described [26].

### 3.2. Antibodies and Major Reagents

Phospho-PDGFRβ (Tyr 751) mouse mAb (3173), PDGFRβ rabbit mAb (3169), phospho-p38 MAPK (Thr180/Tyr182) (12F8) Rabbit mAb (9215), p38 MAPK (7D6) Rabbit mAb (2371), phospho-p44/42 MAPK (Erk1/2) (Thr202/Tyr204) (197G2) Rabbit mAb (4377), p44/42 MAPK (Erk1/2) (137F5) Rabbit mAb (4695), phospho-SAPK/JNK (Thr183/Tyr185) (81E11) Rabbit mAb (4668) and SAPK/JNK (56G8) Rabbit mAb (9258) were purchased from Cell Signaling Technology (Danvers, MA, USA). Monoclonal mouse anti-BrdU (MS-1058) was purchased from Thermo Scientific (Thermo Scientific, Rockford, IL, USA). Monoclonal mouse anti-glyceraldehyde-3-phosphate dehydrogenase (GAPDH) was purchased from KangChen Bio-tech (KangChen, Shanghai, China). Isotype-matched fluorescein isothyiocyanate (FITC)-conjugated anti-rat IgG1 secondary antibody was purchased from Invitrogen (Camarillo, CA, USA). SalA, lyophilized powder, was supplied by Beijing Union Pharmaceutical Factory (Beijing, China). SalA was dissolved in distilled water to reach appropriate concentrations, stored at 4 °C and used as soon as possible. 4',6-Diamidino-2-phenylindole•2HCl (DAPI) was purchased from BIOMOL (Enzo Life Sciences, New York, NY, USA). 5-Bromo-2'-deoxyuridine (Brdu, B9285) and 1,1,3,3-tetramethoxypropane (108383) were purchased from Sigma-Aldrich (Sigma-Aldrich, St. Louis, MO, USA). Propidium iodide (PI) was purchased from Invitrogen.

### 3.3. Proliferation Assays

#### 3.3.1. Crystal Violet Staining

VSMCs and HUVECs were used and the assay was performed as previously described in detail [27].

#### 3.3.2. BrdU Incorporation Assay

VSMCs were seeded at approximately 30% confluence in confocal dishes. After 24 h, cells were starved in FCS-free medium for next 24 h. Cells were pre-treated with SalA at the concentration range of 0–0.1 μM for 2 h and then treated with PDGF-BB (40 ng/mL) for another 24 h. Five hours before harvesting BrdU was added to the medium (10 μM). Cells were trypsinized, fixed in ice-cold ethanol (70%), treated with HCl (2 M) and subsequently with sodium borate buffer (0.1 M, pH 8.5). After incubation with mouse anti-BrdU antibody and isotype-matched fluorescein isothyiocyanate (FITC)-conjugated anti-rat IgG1 secondary antibody, propidium Iodide (PI) was employed to detect nuclei. Labeled cells were examined under a Zeiss confocal microscope, and images were obtained with the laser scanning confocal microscope LEICA TCS SP2 (Leica, Wetzlar, Germany)

### 3.4. Wound-Healing Assay

Cell migration was also assessed by using wound-healing assays as previously described [28]. Confluent VSMCs (starved for 24 h in FCS-free DMEM; migration/growth medium contained 5 mM hydroxyurea to prevent VSMC proliferation) were used. Four different fields of migration were photographed with a video camera system using the Image Pro Plus 5.1 software (Media Cybernetics, Silver Spring, MD, USA) at the intersection of the previously marked line and the wound edge before and after treatment with PDGF-BB for 24 h. Migration was expressed as the change in covered area.

### 3.5. Western Blot Analysis

Immunoprecipitation and Immunoblotting were performed as previously described [29]. The bands on the films were quantified by Quantityone software (Bio-Rad, Richmond, CA, USA) and normalized to GAPDH as a loading control.

### 3.6. Cell Cycle Progression Analysis

VSMCs were seeded into 100 mm culture dishes at 1 × 10^5^ cells/mL until 70% confluence. The medium was then replaced with serum-free media containing SalA (0–0.1 μM). After incubation for 2 h PDGF-BB (40 ng/mL) was added. Subsequently, cells were incubated for 24 h, trypsinized, and then centrifuged at 1,500 g for 5 min. The obtained pellets were suspended in 1 mL of 1 × PBS, washed twice, and re-centrifuged. The pellets were suspended in 70% ethanol and fixed overnight at 4 °C. The cell cycle phase was determined as previously described [30].The proportion of cell in G0/G1, S and G2/M hases were determined using the computer program odFitLT (Verity Software House, Topsham, ME, USA).

### 3.7. Assessment of NO, ICAM-1, VCAM-1 and OPN Production

VSMCs were seeded into 24-well culture plates at 5 × 10^4^ cells/mL until 70% confluence. After incubation for 2 h, PDGF-BB (40 ng/mL) was added, and cells were incubated for 12 h. Supernatants were then collected to determine the levels of ICAM-1, VCAM-1 and OPN detection. Crystal violet staining was used to detect cell number. HUVECs were seeded into 24-well culture plates at 1 × 10^5^ cells/mL until 70% confluence. After synchronized by serum deprivation for 24 h, VSMCs were incubated with Sal for another 24 h. Supernatants were then collected to determine the levels of NO detection. Crystal violet staining was used to detect cell number.

NO levels were determined using the Greiss reagent. The absorbance of the mixture was measured at 595 nm using a SpectraMax M5 plate reader (Molecular Devices, Sunnyvale, CA, USA). The levels of each cytokine were evaluated by using enzyme-linked immunosorbent assay (ELISA) kits according to the manufacturer’s recommendations (R&D Systems, Minneapolis, MN, USA) and the concentration was adjusted according to cell number.

### 3.8. Statistics

Results are expressed as mean ± SEM. For the *in vitro* experiments, data were evaluated by one-way analysis of variance (ANOVA) and the Newman-Keuls post-test. *P* ≤ 0.05 was considered as statistically significant.

## 4. Conclusions

In summary, this study provides the first evidence that SalA strongly inhibits VSMC migration and proliferation induced by PDGF-BB. The inhibition of VSMC proliferation by SalA is associated with cell cycle arrest. Moreover, SalA does not influence endothelial cell proliferation and NO synthesis thereby indicating that it might not interfere with re-endothelialization. Since salvianolic extracts represents an established and safe drug, this could open a novel adjunct pharmacological strategy for the prevention of restenosis.
